# Developing an Ear Health Intervention for Rural Community Pharmacy: Application of the PRECEDE-PROCEED Model

**DOI:** 10.3390/ijerph18126456

**Published:** 2021-06-15

**Authors:** Selina Taylor, Alice Cairns, Beverley Glass

**Affiliations:** 1 Centre for Rural and Remote Health—Mount Isa, 100 Joan Street, Mount Isa 4825, Australia; 2 Centre for Rural and Remote Health—Weipa, 407 John Evans Drive, Trunding 4874, Australia; alice.cairns@jcu.edu.au; 3 Pharmacy College of Medicine and Dentistry, James Cook University, Townsville 4811, Australia; beverley.glass@jcu.edu.au

**Keywords:** expanded practice, scope of practice, models of care, rural health workforce, extended primary healthcare, pharmacist

## Abstract

Unaddressed hearing loss affects an estimated 466 million people worldwide, costing over $750 billion globally, with rural communities being particularly disadvantaged, due to the greater inequity in access to healthcare services. This mixed-methods study aimed to use the PRECEDE-PROCEED model to develop and pilot a rural community pharmacy-based ear health service, LISTEN UP (**L**ocally **I**ntegrated **S**creening and **T**esting **E**ar a**N**d a**U**ral **P**rogram). The PRECEDE process involved an assessment of the predisposing, reinforcing and enabling constructs to support practice change through a scoping review, stakeholder surveys and interviews and consultation with governing bodies and regulatory authorities. The PROCEED segment structured the evaluation of the service pilot and informed planned implementation, process, impact and outcome evaluation. The pilot study conducted in February 2021 included 20 participants, with the most common ear complaints presented being pain, pressure or blockage. All these participants reported high levels of satisfaction with the service, would recommend the service to others and would attend the pharmacy first before seeing a GP for future ear complaints. The PRECEDE-PROCEED model provides a comprehensive model to guide the design of the LISTEN UP program, an innovative model, expanding services offered by rural community pharmacies, with preliminary results demonstrating high consumer satisfaction.

## 1. Introduction

The human ear is an extraordinary organ with intricate anatomy and complex physiology [1]. Its role in hearing, communication and balance is fundamental and disruptions to ear health can significantly impair a person’s function and ability to engage with their environment [1]. Globally, hearing loss is estimated to affect 466 million people worldwide, resulting in a loss of communicative ability, social isolation, loneliness and frustration [2]. Of the 34 million children worldwide with deafness or hearing loss, 60% are a result of preventable causes [2]. The global costs of unaddressed hearing loss are estimated to be $750 billion [2]. Furthermore, the burden of hearing loss is increasing with 900 million people projected to have disabling hearing loss by 2050 [2].

In Australia, one in six people experience some form of hearing impairment with 1.3 million reportedly experiencing preventable hearing loss [3,4]. Conductive hearing loss in Aboriginal and Torres Strait Islander peoples is a major public health problem, with rates as high as 90% reported in some remote communities [5,6]. Australian Indigenous children experience otitis media (OM) younger, more frequently, more severely and for longer durations than non-Indigenous children with the consequences of poorer educational, social and behavioural outcomes, and disrupted connection to land, culture and community [5]. Early detection and intervention, particularly for children, has been recognised as important to improve development and educational outcomes [2].

The main barrier to universal ear and hearing health care is the lack of access to appropriately trained health professionals in low resource communities internationally and within Australia [5]. The limited availability of ear nose and throat (ENT) specialists (78% of global population have <1 ENT per million population), audiologists (93% have <1 per million population) and speech pathologists (83% have <1 per million population) is a major contributor to the global service gap in health system capacity for ear services [7]. Expanding community pharmacists’ roles to support the screening and early identification of ear and hearing complaints should thus be considered to improve the capacity to deliver these services in rural communities.

To date, rural pharmacies have had limited involvement in ear health interventions including screening services (3), otoscopy pilot studies (2), audiometry services (1) and pharmacy-based clinics (3) [8]. These global studies identified in a scoping review have highlighted the potential for pharmacists to expand their scope of practice to address gaps in ear health services for rural and remote communities [8]. Globally, pharmacists are working in expanded roles to better address health needs by providing professional services in addition to traditional medication supply [9]. In Australia, this opportunity for pharmacists to work to their full scope of practice has been limited [10]. This is despite the that fact that community pharmacists as health providers are accessible and have the potential to close the gap on ear disease, and to be better utilised to meet rural community health needs.

This study used the PRECEDE-PROCEED model (PPM) [11] to guide the planning, implementing and evaluation of LISTEN UP (**L**ocally **I**ntegrated **S**creening and **T**esting **E**ar a**N**d a**U**ral **P**rogram), a population ear health program delivered by community pharmacists in two remote communities in Australia [12].

## 2. Methods

### 2.1. Study Design

**Figure 1:**
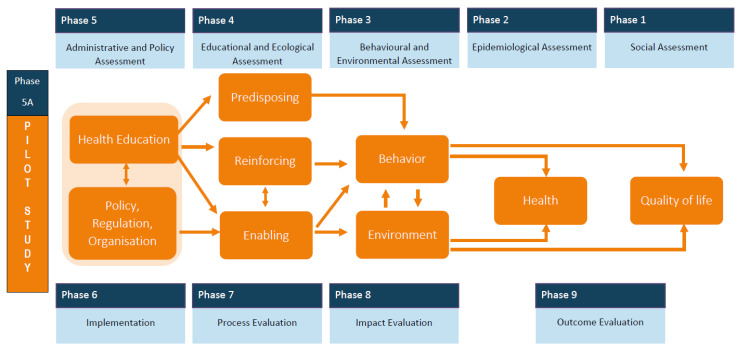
Generic representation of the PRECEDE-PROCEED Model Source: Green and Kreuter, 1999, p. 34 [11].

This mixed-method study applied the PRECEDE-PROCEED Model [12] to plan and develop an ear health program (Figure 1) [13]. Models that support the translation of evidence into practice are known to improve implementation of community health programs [13]. In addition, the PPM has a focus on health promotion, which is a core role for community pharmacists [13]. For the pharmacy profession, a transformation to meet patient-centred care is occurring and the importance of using a model to ensure interventions can be adopted and integrated into clinical and community settings to improve patient outcomes and benefit population health is vital [14].

The PPM has two distinct components, the first, PRECEDE (Predisposing, Reinforcing, and Enabling Constructs in Educational/Ecological Diagnosis and Evaluation) provides an outline of a planning process to guide the development of locally relevant and focused public health programs [11]. The second phase, PROCEED (Policy, Regulatory, and Organisational Constructs in Educational and Environmental Development) provides structure to implement and evaluate the intervention developed in the previous PRECEDE segment [11]. The focus of this paper is on the first five phases of the nine-phase planning model. The fifth phase situated in the PROCEED component was adapted to include a pilot study, which allowed the developed ear health program in the PRECEDE segment to be trialled before implementing and evaluating the final model.

### 2.2. Ethics Approval

James Cook University Human Research Ethics Committee granted ethical approval (H7845 and H8187) for the study.

#### PRECEDE Planning Model Component

Scoping and systematic reviews and studies including both qualitative and quantitative methods, involving community consultation with the public and health professionals, who work in rural and remote locations in Australia were undertaken in this component of the application [8,15,16,17,18,19,20,21], Table 1 provides the components of the PRECEDE-PROCEED model in the context of LISTEN UP.

### 2.3. Intervention Context and Setting

LISTEN UP is being delivered by two rural community pharmacies in Queensland, Australia. The first site (population 18,000; 17% Indigenous population; Modified Monash Model (MMM) category 5—remote community) is a remote mining town situated 1000 km from tertiary medical facilities and 1800 km from a capital city [22,23]. It is a town with three community pharmacies, three GP practices and a small 80-bed hospital. The second site (population 6000; 6% Indigenous population; MMM category 4—medium rural town) is a rural agricultural town situated 350 km from a capital city and tertiary medical facilities [22,23]. It is serviced by a small 32-bed hospital, one GP practice and two community pharmacies [22,23].

### 2.4. Procedure and Participants

Phase 1–5: Data from the literature and research were synthesised and disseminated to participating pharmacies during formal meetings to discuss and inform the development of the model. This information was also shared with the advisory panel which was formed by inviting interested stakeholders, governing bodies, regulatory authorities and community organisation representatives to participate in the pilot study in an advisory capacity. This provided an opportunity to ensure the model would fit the pharmacy, community and profession.

Pilot Study: Patients aged >13 years presenting to the participating pharmacies with an ear complaint were invited to receive the LISTEN UP service. Pharmacists (with additional training) conducted an examination including a brief history, hearing screening, otoscopy and tympanometry assessments following a study protocol (Figure 1) [24], and patients were recommended no treatment, pharmacy only treatment or referral to a general practitioner (GP) if required [24].

### 2.5. Data Collection

Phase 1–5: Data collected included questionnaires completed by stakeholders including consumers, pharmacists and health professionals [16,17,18]. Expanded pharmacy services were ranked by participants, the level of support for expanded pharmacy services was explored and consumers’ willingness to pay for services examined [16,17,18]. Interviews were conducted with pharmacists and stakeholders to further explore perceptions, and barriers and enablers to expanded pharmacy services, specifically relating to ear services [20,21].

Pilot Study: Pharmacists recorded patient data on a service summary record and a GP referral template. Patients also completed a satisfaction survey and received a follow up phone call at seven days, which was transcribed and analysed to explore outcomes including prescribed medications and referrals [24]. The LISTEN UP satisfaction survey was based on a survey for a sore throat study in the UK, where pharmacists are delivering consultations including clinical scoring and point-of-care testing [25].

### 2.6. Data Analysis

A descriptive analysis of questionnaire data, a thematic analysis of interview transcripts and a review of consultation data were undertaken [16,17,18,19,20,21].

## 3. Results

### 3.1. PRECEDE

The results of the PRECEDE component of the planning model are presented and summarised in Table 2.

#### 3.1.1. PRECEDE—Phase 1—Social Assessment

A social assessment of rural consumers, pharmacists and health professionals was undertaken. During this phase, data were collected via questionnaires that enabled participants to rank the importance of an ear health strategy for rural community pharmacies. Overall, hearing tests were ranked as the seventh (from twenty-six services) most important expanded pharmacy service by health professionals and consumers [16,17]. Although other expanded services were ranked more highly, discussions with rural pharmacists identified ear heath as an unmet healthcare need in their populations. In addition, there was recognition of the profound impact of unmanaged ear conditions on quality of life for both children and adults, and particularly for Indigenous populations [5,7].

#### 3.1.2. PRECEDE—Phase 2—Epidemiological Assessment

A systematic [15] and scoping review [8] and interviews [19,20] were undertaken to gather knowledge of expanded pharmacy services broadly, expanded services in rural pharmacy settings and pharmacy ear services. There were a limited number of articles found in both the reviews, however findings from the reviews informed the design of the interview question guides. Discussions of the challenges and opportunities in rural pharmacy practice broadly and specifically for an ear service were undertaken during the interviews. Questions were asked of community pharmacists and experts in the ear, nose and throat specialty about what problems affect the ear health of the community, what needs to change to improve ear care and what role community pharmacists can play in ear care. The findings were used to develop two primary objectives of the 12-month ear health pilot study:

- To improve rural consumer access to ear health care; and
- To determine pharmacist level of preparedness and confidence to examine an ear and make appropriate recommendations or referrals following a protocol.

#### 3.1.3. PRECEDE—Phase 3—Behavioural and Environmental Assessment

A scoping review revealed limited research into ear health models provided in community pharmacy (no Australian studies) with some studies reporting hearing testing being provided in community pharmacies by external audiology services in Australia [8]. Previous research identified key behaviours of people attending community pharmacy for ear complaints. It was found that ear complaints are common in children, in which case pharmacists regularly provide pain management options and recommend referral to general practice and more commonly to emergency departments, due to the lengthy wait for a doctor’s appointment [20].

Interviews with pharmacists identified instances where pharmacists, particularly those working in coastal regions, are providing an informal ear care service and using otoscopy to examine patients’ ears [20]. In addition, discussions about private consultation rooms, workload difficulties particularly for sole pharmacists and remuneration considerations were held [20]. Interviews with health professionals highlighted concerns about the role of the pharmacist and pharmacists’ skills and knowledge to diagnose or treat ear conditions [20]. Consequently, the pilot study protocol was developed without the pharmacists diagnosing or treating any ear conditions that they would not usually manage and instead requires the pharmacists to provide, for data collection purposes only, a clinical impression of the condition and describe what treatment or referral they would make if there were no restrictions [24].

Following this phase of the assessment, the secondary objectives of the ear health strategy were developed:

- To identify untreated ear conditions in rural communities, which may lead to reduced complications, developmental delay and functional impairment;
- To improve collaboration between community pharmacy and general practitioner (GP) services;
- To provide targeted patient ear health referrals to GP practice; and
- To support engagement of telehealth through the use of video-otoscopy and timely transfer of care.

#### 3.1.4. PRECEDE—Phase 4—Educational and Ecological Assessment

We identified potential factors that would need to be modified to effect change in ear care services in community pharmacy. These factors were identified based on discussions with stakeholders, rural pharmacists and community leaders, recent research and a review of the literature [8,15,18,19,20]. Existing consumer outcome expectations when presenting to community pharmacies with an ear complaint were identified as either a product recommendation or verbal referral to a GP or emergency department. Similarly, pharmacists expected the same outcomes when consulting a patient with an ear complaint.

Training was a major consideration in developing the intervention. Ear, nose and throat specialists were consulted on the skills in otoscopy and tympanometry that were required for pharmacists to effectively conduct an ear examination. Consequently, each participating pharmacist undertook nationally credentialed training in ear health. This training was delivered by mixed mode with online and face-to-face components and included the development of skills in otoscopy and tympanometry [26]. The training includes 55 h of online training and 16 h of workshops [26]. The training delivered by Benchmarque group was a purposely designed one-off hybrid of other ear education training programs (including training for nurses, doctors and Indigenous health workers) [26]. Topic areas included foundations of ear health including anatomy and physiology, theoretical and practical sessions on ear condition recognition and assessment, as well otoscopy and tympanometry skills [26]. The following competencies were part of the training units: EHHPEH002—Promote, educate and manage ear health, EHHAEH001—Assess ear health, EHHPEA004—Paediatric and TYMPTY001—Perform Tympanometry [26].

Reinforcing a behaviour is an important construct in social cognitive theory and it requires a behaviour to be repeated and sustained [27]. As this service is focused on acute presentations of ear complaints to a community pharmacy, the reinforcing factor is that this service was implemented as a pilot service and will continue as a permanent service after the pilot concludes. The pharmacies will retain the equipment and thus be able to continue to offer the service and sustain the ear care focus for their communities. In addition, behaviour change through the non-pharmacological advice and health promotion provided during the service is a reinforcing factor of the model.

#### 3.1.5. PRECEDE—Phase 5—Administrative and Policy Assessment

Based on the analysis of assessments in Phases 1–3, we constructed a preliminary ear health intervention for the rural community pharmacy. Through consultation with the two community pharmacists involved in the study, the capacity of the pharmacies to implement an ear health intervention was explored. Both pharmacies reported adequate pharmacists, time and space for the project. The primary capital expense was the purchase of an equipment package including a video-otoscope, tympanometer and consumables for each pharmacy. Funding was also required for the training.

An advisory panel was then formed with representatives from key stakeholder organisations including Pharmaceutical Society of Australia, Pharmacy Guild of Australia, Gidgee Healing (Aboriginal Medical Service), and Australian Primary Health Network. Consultations were conducted and minor changes such as wording on documentation were implemented. The advisory panel provided expertise to this project and confidence that the study was appropriate and acceptable in practice and well aligned with the current rural pharmacy landscape. General practitioner engagement followed, whereby GP practices were invited to participate in the project. In each site, a GP service agreed to participate and their contribution to the project was to provide the link between the community pharmacy consultations and GP presentations. Participants who were seen at the pharmacy and required a GP referral were connected to the GP service with a same-day or next-day appointment.

Policy, regulation and legal requirement were discussed through consultations with representatives from Pharmaceutical Society of Australia, Pharmacy Guild of Australia, The Pharmacy Board of Australia, Pharmaceutical Defence Limited and the Department of Health. It was agreed that a pharmacist’s scope of practice is determined by an individual pharmacist and with the proposed training, the LISTEN UP service was within the pharmacist’s role with respect to both regulation and the law [28].

### 3.2. PROCEED

**Figure 2:**
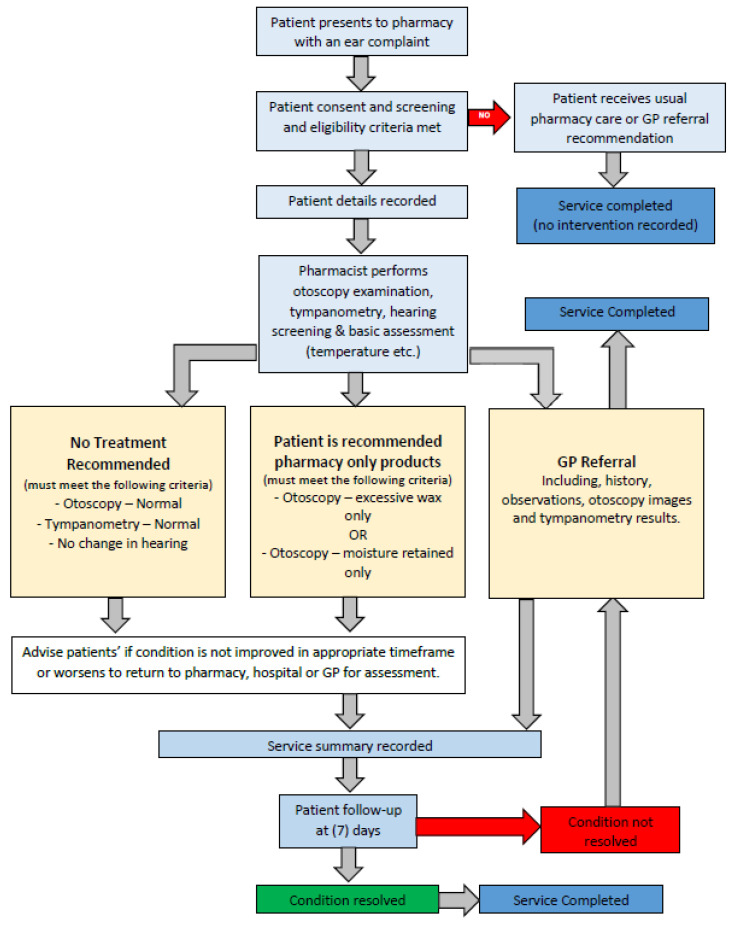
Study Protocol for LISTEN UP [24].

The study protocol for LISTEN UP is registered with the Australian and New Zealand Clinical Trial Registry: ACTRN12620001297910 (Figure 2). This project has been approved by the Human Research Ethics Committee, James Cook University. (Reference number: H8187). A detailed study protocol has been published in the Pilot and Feasibility Studies Journal [24]

#### 3.2.1. Phase 5A—Pilot Study

The pilot study started on 1 February 2021. During the six-week pilot, 20 participants presented to the pharmacy with an ear complaint. One participant had a fever within 72 h and was excluded from the trial and one participant’s data record was incomplete and thus excluded. The demographic and clinical data from the pilot study are reported in Table 3. The average age was 44 years with a range from 20 to 71 years. Two-thirds of the participants were female. Sixteen of the participants were able to be contacted for a follow-up interview, with eleven identified as Caucasian and five as Aboriginal or Torres Strait Islander.

Otoscopy examinations occurred in 16 of the consultations with 12 reported by pharmacists as normal. Tympanometry assessments were conducted for 14 participants and 10 were reported as normal by the pharmacists. Pharmacist recommended products included wax dissolvents (*n* = 7) or analgesic therapy (*n* = 3) and four participants were not recommended any treatment. Four patients were referred to and attended GP appointments. At the seven day follow up five participants symptoms had completely resolved, three were improving, one was not improving, and this participant was referred to the GP. One participant who was recommended no treatment had attended the emergency department at the hospital and no treatment was recommended. Of the four participants who attended the GP, two cases were resolved and two were not and those unresolved cases had follow up appointments in place with the GP. Four participants were unable to be contacted for the follow up.

The patient satisfaction results are provided in Table 4. All participants agreed or strongly agreed that the pharmacist explained the aims of the LISTEN UP service well, that they were satisfied with how the pharmacist checked their ears and recommended treatment options, that they had the opportunity to raise questions or concerns relating to the service and that they would recommend the LISTEN UP service to others. Participant satisfaction themes were focused on convenience, timeliness, professionalism and knowledge of pharmacists. All participants stated they would pay for the service in the future and the value ranged from AUD$15–$200.

#### 3.2.2. Phase 6—Implementation

Program implementation commenced March 2021.

#### 3.2.3. Phase 7—Process Evaluation

The process evaluation was conducted upon the completion of Phase 5A—Pilot Study. In addition to the results provided, discussions were held with the pharmacists about the program and any improvements that were needed. Both pharmacies had the same equipment availability, a similar number of pharmacists employed and a strong dedication to professional service provision. However, only one of the participating pharmacies had begun the program, and the other reported that difficulties such as workload, hesitation around the documentation processes and other competing business priorities which had been impacting on their ability to commence LISTEN UP. Additional support through site visits has been offered to this pharmacy to facilitate the implementation of LISTEN UP.

For the pharmacy that participated in the pilot, two of the pharmacists conducted all the consultations and those two pharmacists dedicated time to focus on over-the-counter requests. Barriers reported by the pharmacists included difficulty in providing the consultation in a timely manner that allowed for documentation to be completed, frustration at needing to refer patients to a GP for conditions that could be managed in the pharmacy, and not being remunerated for the service. Pharmacists expected that if their scope of practice expanded to include prescribing for minor ailments, LISTEN UP would save both time and money. Consideration for future research would include having dedicated face-to-face implementation sessions at every site to ensure all pharmacists are confident to begin the program on completion of the training.

There were also initial reports of difficulty in making patients timely appointments at the GP practice. Consultation with the GP practice identified scheduling difficulties being associated with extended waiting times for GP appointments, and the GP practice was then able to prioritise the referrals from the pharmacies to ensure the project could maintain fidelity.

To determine the efficacy of the LISTEN UP program approximately 200 participants will be required to partake in the service for an appropriate sample size [24]. This will allow for measures including intervention exposure, fidelity and participant appraisal to be examined on a robust volume of data. As there were minimal changes incorporated after the six-week pilot study, the data from the pilot will be included in the twelve month pilot evaluation.

## 4. Discussion

In Australia, around 3.6 million people suffer from hearing loss, with more than 1.3 million hearing conditions that could have been prevented [4]. When left untreated, hearing loss and ear disease can affect a child’s learning and development and those with untreated hearing loss may also be at risk of developing other health problems [4]. Barriers to accessing ear care services have been identified including gaps in testing during the ‘early years’ and difficulty in accessing health services [29]. In rural and remote populations, the burden of ear disease is under-reported with widely recognised and profoundly negative impacts on patient outcomes, particularly for Indigenous people [3]. Rural community pharmacists have been identified as highly qualified and easily accessible health professionals in rural and remote communities [10]. However, data for patients presenting with ear complaints to rural or remote community pharmacies is unavailable. In many rural communities, Indigenous populations can be as high as 20–80% and thus the likely prevalence of hearing issues would be significant. Thus, a paradigm shift of the role of rural community pharmacists to provide an ear health program that has the potential to improve health outcomes for rural and remote populations.

The lack of literature combined with the knowledge of pharmacists providing unregulated ear care services highlights the importance of developing a structured ear care model for rural community pharmacy and the pharmacy profession more broadly. Historically pharmacists have provided advice at no cost and this has been recognized as a barrier to pharmacists being able to provide sustainable professional services [30]. There is an expectation that consumers would not be willing to pay for pharmacy services, and thus either a fee for service model whereby consumers pay or by attracting government funding if the model were found to be successful would improve the sustainability of LISTEN UP [30].

Another enabling factor identified in the literature was high quality training for the pharmacists providing the service [8]. We attracted approximately AUD$20,000 in funding to provide a training and equipment package for pharmacists in two rural community pharmacies and discussed the opportunity with the participating pharmacists to continue to provide a financially viable service after the intervention period is completed. The importance of ensuring that pharmacists providing professional services are well trained to do so has been identified by pharmacists themselves who described concern about their skills for expanded services [20]. This was also the opinion of other health professionals who were largely supportive of pharmacists providing expanded services as long as adequate training has been provided [17,18,21].

The LISTEN UP pilot study has demonstrated very high levels of patient satisfaction and intention to return to the pharmacy for ear complaints in the future. This satisfaction level aligns with the sore throat study conducted in the United Kingdom, which may indicate the beginning of a shift in consumer health-seeking behaviour towards pharmacy-based services [25].

Difficulty with incorporating a new service into the workflow and managing the additional workload for professional services in pharmacy has been reported in the literature [15,31]. Pharmacist availability and additional time required to complete documentation associated with services have been described as barriers to service delivery [15,31]. LISTEN UP has been successfully implemented into a pharmacy that operates with the equivalent of 4 full-time pharmacists, with a strong focus on professional services. The pharmacy has a dedicated pharmacist providing professional services during all operating hours and this behavioural change and focused time for service provision has seen LISTEN UP easily implemented as part of the pharmacy’s suite of professional services.

The discussion and collaboration with the GPs before the pilot and agreement by them to facilitate timely appointments has been crucial to the program. In other studies, a lack of collaboration from GP providers has been reported as a major barrier to the success of professional pharmacy services [32,33,34]. GPs have been described as unsupportive of pharmacy services broadly and although GPs have been reported to be accepting of pharmacists’ involvement in medication management, they were less accepting of expanded services [15,34,35].

### 4.1. Phase 8—Impact Evaluation

Findings from the clinical data of patient presentations will demonstrate if the program is effectively identifying untreated ear conditions. In addition, the follow up phone call to determine if the conditions have resolved will determine if the program is effectively managing ear conditions to reduce complications, developmental delay and functional impairment.

Interviews with pharmacists and GPs pre- and post-intervention will determine whether the project has improved collaboration between community pharmacists and GPs. GP perspectives as to whether the protocol is ensuring they are receiving targeted referrals and whether the utilisation of telehealth and video-otoscopy is resulting in timely transfer of care will be identified in the interviews with the GPs post the intervention period.

Pharmacy-based referrals to GPs and telehealth services with GPs offered through pharmacy have been reported previously in the literature. In a study about GPs’ (*n* = 414) attitudes towards minor ailment management, there is an agreement (77%) that patients should visit a pharmacist about minor ailments before visiting the doctor and that the pharmacists’ role is to provide advice on appropriate course of action with referral to GP if necessary (90%) [36]. GPs are also offering telehealth services for patients from within the community pharmacy, further strengthening a link between GP and pharmacy and optimizing a continuity of care for patients [37].

### 4.2. Phase 9—Outcome Evaluation

The outcome evaluation will include data from patient satisfaction surveys, patient follow-up phone call transcripts, consultation data and interview transcripts from pre and post interviews with pharmacists and GPs. This data will be evaluated to determine if the LISTEN UP program has improved rural consumer access to ear health care and whether pharmacists are prepared and confident to provide an ear health service following a protocol.

### 4.3. Limitations

This study is limited due to sample size and number of participating pharmacies. The sample size notwithstanding, this pilot service is the first attempt to improve ear health of rural populations who are extremely vulnerable due to lack of access to appropriate services. The qualitative component of the study has also allowed for a deep understanding of challenges and enablers to developing and implementing an ear health service.

## 5. Conclusions

The impact of ear disease in rural and remote communities is profound. The extreme lack of access to health providers working in ear health and increased severity of ear disease will continue to impact education, employment and social opportunities. The PRECEDE-PROCEED model can be effectively applied to undertake a stepwise approach to design, develop and evaluate an innovative model of care. LISTEN UP is a community pharmacy ear program that has been piloted in two remote Australian communities with promising results, reflected in the highly positive consumer feedback. Although difficulties have arisen with implementation across more than one site, the PRECEDE-PROCEED model may be used to identify implementation issues earlier before major investment has occurred. Successful application of the PRECEDE-PROCEED model to the LISTEN UP may confirm its usefulness in the development of other services where gaps in existing service provision have been identified.

## Figures and Tables

**Table 1:** Components of the PRECEDE-PROCEED model in the context of LISTEN UP.

Construct	Definition as Applied to This Study	Data Source
Phase 1—Social Assessment	Determine desired outcomes and goals of LISTEN UP for rural consumers, pharmacists and health professionals.	Questionnaire and interviews [16,17,18].
Phase 2—Epidemiological Assessment	Determine measurable, time-limited, health-related objectives of LISTEN UP.	Systematic review [15], scoping review [8] and interviews [19,20].
Phase 3—Behavioural and Environmental Assessment	Identify key environmental and behavioural factors that may impact or influence LISTEN UP. Develop sub-objectives of LISTEN UP.	Systematic review [15], scoping review [8] and interviews [19,20].
Phase 4—Educational and Ecological Assessment	Determine modifiable factors (predisposing, enabling and reinforcing) that would result in behaviour change and a sustainable change process.	Systematic review [15], scoping review [8] and community consultation.
Phase 5—Administrative and Policy Assessment	Investigation and application of policy, regulation and law surrounding community pharmacy practice.	Consultation with policy makers, regulatory and governing bodies.
Phase 5A—Pilot Study	Pilot the intervention for six months.	Pilot study data and consultation with pharmacists.
Phase 6—Implementation	Implementation of the intervention for six to twelve months.	
Phase 7—Process evaluation	Assessment of the intervention exposure, the extent to which the program is implemented as designed and participant appraisal of the intervention.	Participant demographic data. Participant satisfaction survey. Pharmacist interviews on feasibility, barriers and enablers to intervention implementation.
Phase 8—Impact evaluation	Assessment of the behavioural and environmental sub-objectives by identifying untreated ear conditions in the community, improving collaboration with GPs through targeted referrals and utilisation of telehealth technologies.	LISTEN UP complete data set. Pre- and post- interviews with GPs and pharmacists.
Phase 9—Outcome evaluation	Assessment of public health impact through exploration of patient experience of accessing ear care.	LISTEN UP complete data set. Pre- and post- interviews with GPs and pharmacists.

**Table 2:** PRECEDE Results with linkage to data source.

Data Source	Related Phase	Results Relevant to Model
Systematic review [15]	Behavioural and environmental assessment	Limited expanded service models in rural pharmacy practice. No ear services identified. Barriers and enablers explored and considered for LISTEN UP model.
Scoping review [8]	Behavioural and environmental assessment	Limited pharmacy ear health services in community pharmacy. Barriers and enablers explored and considered for LISTEN UP model.
Consumer questionnaires [16]	Social assessment	Hearing health ranked seventh of 26 expanded pharmacy services.
Health professional questionnaires and interviews [17,19,21]	Social assessment	Hearing health ranked seventh of 26 expanded pharmacy services. Varying levels of support for pharmacists to provide expanded services depending on profession and location.
Pharmacist questionnaires and interviews [18,19]	Social assessment Educational and ecological assessment	Expected improved health outcomes and increased access from pharmacists providing expanded services. Consensus that the management of ear health in pharmacy could be improved.
Stakeholder interviews [19] including: Pharmaceutical Society of Australia, Pharmacy Guild of Australia, Aboriginal Medical Service, and Australian Primary Health Network	Educational and ecological assessment	Consensus for rural pharmacists to increase service delivery for ear care.
Policy and regulatory meetings	Administrative and policy assessment	Administrative requirements, indemnity insurance, scope of practice and training incorporated into model.
Specialist health professional interviews	Educational and ecological assessment	Training and education recommendations and best practice suggestions to be incorporated into model.
Advisory panel	Educational and ecological assessment Administrative and policy assessment Continuing through PROCEED segment.	Positive response to the model with minor suggestions incorporated.

**Table 3:** Patient demographic and clinical data (*n* = 18).

Age (Years)		Gender	
19–24	3 (17%)	Female	12 (67%)
25–34	5 (28%)	Male	6 (33%)
35–44	1 (5%)	Ethnicity	
45–54	3 (17%)	Caucasian	11 (61%)
55–64	4 (22%)	Indigenous	5 (28%)
65 and above	2 (11%)	Unknown	2 (11%)
Presenting Complaint (*n* = number of patients)		Pharmacist’s Clinical Impression (number of patients)	
Pain/Pressure	11	Ear wax impaction	6
Blocked (wax/water)	10	Ruptured ear drum	3
Hearing Impairment	3	Unsure	3
Itch	2	Normal ear	2
Other	2	Inflammation	2
		Calcification	2
		Otitis media	2

**Table 4:** Patient satisfaction survey results.

Questions with Yes/No Answer Option	Yes
Before coming to the pharmacy today, I tried to see a GP about my ear	6 (33%)
If the service was not available today I would have gone to my GP	13(72%)
If the service was not available today I would have gone to the hospital	10 (50%)
Next time I have an ear problem I will come to the pharmacy instead of a GP	18 (100%)
Free Text Comments	
“Very good reassurance about my ears.” “Service exceeded my expectation.” “I am satisfied with how the pharmacist checked my ears. Great service.” “Excellent support, information was great, feel reassured. Thank you.”	

## Data Availability

The data presented in this study are available upon request from the corresponding author. The data are not publicly available as the trial is currently in progress.

## Clinical Trials Registration

LISTEN UP is registered with the Australian New Zealand Clinical Trials Registry (ANZCTR). ACTRN: ACTRN12620001297910.
